# The load-velocity relationship in the jump squat exercise

**DOI:** 10.5114/biolsport.2023.118019

**Published:** 2022-09-06

**Authors:** Irineu Loturco, Michael R. McGuigan, Lucas A. Pereira, Fernando Pareja-Blanco

**Affiliations:** 1 NAR – Nucleus of High Performance in Sport, São Paulo, Brazil; 2 Department of Human Movement Sciences, Federal University of São Paulo, São Paulo, Brazil; 3 University of South Wales, Pontypridd, Wales, United Kingdom; 4 Sports Performance Research Institute New Zealand (SPRINZ), Auckland University of Technology, Auckland, New Zealand; 5 School of Medical and Health Sciences, Edith Cowan University, Perth, Australia; 6 Physical Performance & Sports Research Center, Pablo de Olavide University, Seville, Spain

**Keywords:** Athletic performance, Athletes, Muscle strength, Resistance training, Team sports, Loaded jumps

## Abstract

The purpose of this study was to test the load-velocity relationship in the jump squat (JS) exercise using three different velocity parameters (mean velocity [MV], mean propulsive velocity [MPV], and peak velocity [PV]). Twenty-six male rugby union players (24.3 ± 3.9 years; 1.81 ± 0.09 m; 101.3 ± 15.4 kg) performed a progressive loading test in the JS with loads corresponding to 20, 40, 60, and 80% of the half-squat 1RM (equivalent to 24, 46, 70, and 94% of the estimated JS-1RM). MV, MPV, and PV were continuously recorded during all attempts using a linear velocity transducer. Linear regression models were used to determine the relationships between JS loads and MV, MPV, and PV. Bar-velocity outputs demonstrated high levels of consistency and reliability (coefficient of variation ≤ 5% and intraclass correlation coefficient ≥ 0.90). The predictive power of MV, MPV, and PV were ≥ 91%, for all tested variables (P < 0.0001). The equations and bar-velocity values provided in this study can be used by coaches to precisely determine and prescribe JS training loads, from verylight to heavy loading conditions (i.e., ~20–100% JS 1RM).

## INTRODUCTION

Squatting variations are undoubtedly the most frequently used exercises in a wide variety of sport disciplines [1–3]. Whereas “traditional squats” (e.g., front-, half-, or full-squat) are often implemented to develop strength-related qualities (e.g., maximum strength), the ballistic jump squat (JS) is primarily used to increase the ability to apply force at higher velocities (i.e., muscle power) [4–7]. The JS is usually performed with light or moderate loads (i.e., 30–45% of one-repetition maximum [1RM]), prescribed on the basis of the halfsquat 1RM (HS-1RM) [4, 8]. Nevertheless, despite their similarities, it is worth noting that HS and JS are different exercises, as the JS can only be executed when a “valid” jump attempt is possible [4, 8].

A recent study sheds light on these mechanical differences, by analyzing the kinematics of the HS exercise during an incremental loading test [8]. In brief, it was observed that at 86% HS-1RM the concentric portion of the lift is entirely propulsive, which means that the athlete does not have to gradually decelerate across the movement in order to stop the barbell [8]. This occurs because the barvelocity at this load condition is very low (i.e., ≤ 0.4 m · s^−1^), thus preventing the existence of a “braking phase” (i.e., a phase where the force applied by the athlete against the external load is negative) at the end of the lift [8, 9]. From a mechanical perspective, at 86% HS-1RM it is impossible to jump. As a consequence, this relative strength value should be used as a reference for the 1RM in the JS (JS-1RM) [8]. Developing approaches to determine the JS-1RM in a more practical manner (instead of using the time-consuming and demanding HS-1RM measurement) would be relevant for coaches and sport scientists, especially those working in high-performance environments, where time-constraints are a factor.

The load-velocity relationship has been widely used by practitioners to predict loading intensity in several upper- and lower-body exercises [10–14]. A seminal study on the topic stated that “the inextricable relationship that exists between relative load (%1RM) and movement velocity” allows coaches to rapidly determine the 1RM load and the %1RM used with great precision and accuracy [15]. Despite this solid body of evidence, to date, only one study has examined the load-velocity relationship in ballistic versions of the HS. In that study, the authors found strong relationships between %1RM and bar-velocity, for both loaded squat and countermovement jumps (R² = 0.88 and 0.96, respectively) [11]. However, the research was conducted with sport science students, with %1RM determined on the basis of the HS-1RM and utilizing only the mean propulsive velocity (MPV) in its predictive models. In this context, it is important to analyze this mechanical relationship in highly-trained subjects, implementing the novel JS-1RM approach in a more comprehensive manner (i.e., using simultaneously MPV, mean-velocity [MV], and peak-velocity [PV] measures). This is crucial to provide coaches with more precise information regarding the prescription of loaded jumps for elite athletes. Therefore, the purpose of this study was to test the load-velocity relationship in the JS exercise under different percentages of the JS-1RM.

## MATERIALS AND METHODS

### Participants

Twenty-six male rugby union players from the Brazilian National Team (24.3 ± 3.9 years; 1.81 ± 0.09 m; 101.3 ± 15.4 kg) participated in this study. The study was approved by the local Ethics Committee and all athletes signed an informed consent form prior to participation.

### Study Design

This cross-sectional study was designed to test the load-velocity relationship in the JS exercise. Due to the constant training and testing in our facilities, all athletes were well familiarized with testing procedures. The assessments were performed at the end of the preseason period, over two consecutive days. Prior to the testing session, the rugby players performed standardized warm-up protocols including general (i.e., running at moderate pace for 10-min followed by 3-min dynamic stretching and mobility exercises) and submaximal attempts of JS.

### Procedures

#### Maximum dynamic strength in the half-squat and progressive loading test in the jump-squat exercise

Maximum dynamic strength was assessed in the HS exercise as described previously [16, 17]. Firstly, athletes executed a warm-up set, consisting of 5 repetitions at 50% of the estimated HS-1RM on the Smith-machine device (Hammer-Strength, IL, USA). Subsequently, athletes were allowed up to 5 attempts at approximately 70, 80, 90, and > 95% of the estimated 1RM to obtain the actual HS-1RM value. On the second day, the load-velocity relationship was progressively assessed in the JS exercise, with loads corresponding to 20, 40, 60, and 80% HS-1RM (equivalent to 24, 46, 70, and 94% of the estimated JS-1RM) [8, 16, 17]. For all rugby players, at 80% HS-1RM, the relative contribution of the propulsive phase to the total concentric duration in the HS exercise was ≥ 98% [18]. Athletes were required to perform two repetitions with each relative load and move the barbell as rapidly as possible during the concentric phase of the lift. The measurements were conducted by an experienced researcher who standardized the degree of the knee flexion (i.e., 90° knee angle) through visual inspection [8]. A 3-min rest interval was provided in all trials [8]. MV (mean velocity from the start of the concentric phase until the barbell reaches the maximum displacement), MPV (mean velocity from the propulsive phase), and PV (maximum instantaneous velocity recorded over the entire concentric phase) [19] were continuously measured and recorded at 1000 Hz, using a valid and reliable linear velocity transducer (T-Force System; Ergotech Consulting S.L., Murcia, Spain) attached to the barbell [20, 21].

### Statistical Analyses

Data are presented as mean ± standard deviation. A linear regression was performed to establish the load-velocity relationship in the JS. The standard error of estimate (SEE) and the coefficient of determination (R^2^) were calculated. The level of significance was set at *P* < 0.05. Absolute and relative reliability between repetitions within the testing session were assessed for bar-velocities across the relative loading ranges, using the coefficient of variation (CV) and a 2-way random effects model intraclass correlation coefficient (ICC), respectively.

## RESULTS

**FIG. 1:**
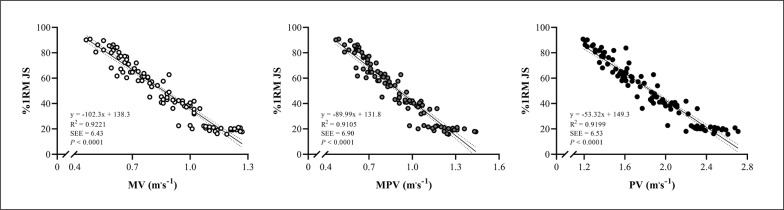
Linear regression models (with 95% confidence limits) representing the relationships between different relative loads (independent variables) and bar-velocity outputs (dependent variables) in the jump squat (JS) exercise. %1RM JS: percentage of one-repetition maximum; MV: mean velocity; MPV: mean propulsive velocity; PV: peak velocity; SEE: standard error of estimate.

Table 1 shows the absolute and relative reliability of the bar-velocities at the different relative loads tested. Figure 1 depicts the relationship between the different percentages of 1RM (independent variable) and bar-velocities (dependent variables). The R² of the distinct load-velocity relationships was 0.92, 0.91, and 0.91 for MV, MPV, and PV, respectively (*P* < 0.0001 for all variables). Table 2 presents the bar-velocities attained at different percentages of 1RM during the JS exercise.

**TABLE 1:** Absolute and relative reliability of bar-velocities across the range of loads in the jump squat exercise.

Relative load (%1RM)	Velocity parameter	CV	95% CI		ICC	95% CI	
			Lower	Upper		Lower	Upper
24% (*20%)	**MV**	2.32	1.34	3.31	0.92	0.82	0.97
	**MPV**	2.41	1.07	3.74	0.91	0.79	0.96
	**PV**	2.06	1.47	2.66	0.94	0.87	0.97

46% (*40%)	**MV**	1.78	1.17	2.39	0.95	0.88	0.98
	**MPV**	2.74	1.87	3.61	0.91	0.8	0.96
	**PV**	1.23	0.86	1.6	0.98	0.96	0.99

70% (*60%)	**MV**	4.49	2.87	6.11	0.94	0.85	0.97
	**MPV**	4.67	2.77	6.58	0.93	0.84	0.97
	**PV**	1.67	1.12	2.23	0.98	0.95	0.99

94% (*80%)	**MV**	4.44	2.89	6	0.9	0.76	0.96
	**MPV**	4.94	3.29	6.59	0.9	0.76	0.96
	**PV**	2.49	1.72	3.26	0.98	0.96	0.99

CV: coefficient of variation; CI: confidence interval: ICC: intraclass correlation coefficient. MV: mean velocity; MPV: mean propulsive velocity; PV: peak velocity; 1RM: one-repetition maximum. *The numbers in brackets indicate the percentages of half-squat 1RM.

**TABLE 2:** Bar-velocities across the range of loads in the jump squat exercise.

%1RM	MV (m · s^−1^)	MPV (m · s^−1^)	PV (m · s^−1^)
24	1.14 ± 0.09	1.23 ± 0.11	2.41 ± 0.16
46	0.94 ± 0.07	1.00 ± 0.08	2.00 ± 0.14
70	0.76 ± 0.08	0.78 ± 0.09	1.66 ± 0.14
94	0.61 ± 0.08	0.63 ± 0.08	1.38 ± 0.13

Data are expressed as mean ± standard deviation. 1RM: one-repetition maximum; MV: mean velocity; MPV: mean propulsive velocity; PV: peak velocity.

## DISCUSSION

We examined the load-velocity relationship in the JS exercise using three bar-velocity outputs (i.e., MV, MPV, and PV) as independent variables. Overall, all these measures exhibited high levels of accuracy and consistency (R² ≥ 0.91, CV ≤ 5%, ICC ≥ 0.90) across the wide range of loads (~20–100% JS-1RM). Therefore, MV, MPV, and PV can be used to predict relative loads in the JS in an interchangeable manner.

For the first time, we adopted the JS-1RM load (i.e., 86% HS-1RM) [8] as the reference for prescribing relative loads in the JS. A previous investigation on the load-velocity relationship in this ballistic exercise also reported a similar shared variance of ~90% between MPV and different percentages of 1RM [11]. Nevertheless, in that study, JS loads were determined based on the traditional HS-1RM approach. In addition, the authors used only the MPV in the linear regressions, thus limiting loading predictions from other barvelocity outputs. Our results confirm and extend these findings, indicating that either MV, MPV, or PV may be used to determine JS loads with high and similar levels of precision (R² = 0.91–0.92). Based on the data provided here, coaches can now prescribe and monitor the JS intensity in a very practical manner, irrespective of their personal preferences (using MV, MPV, or PV) and technical possibilities (i.e., via linear transducers or accelerometers) [22].

Despite the ballistic nature of the JS and the probable influences of distinct mechanical factors on its respective load-velocity relationship (e.g., higher movement velocities, the existence of flight phase, use of mean or peak velocity measures), the predictive power and consistency of the linear equations reported here are similar to those described for other “traditional” (i.e., non-ballistic) exercises (R² ≥ 0.90, CV ≤ 5%, and ICC ≥ 0.90) [11, 18]. Furthermore, the inclusion of very-light relative intensities (i.e., ~20% JS-1RM) in the linear regressions allows coaches to precisely determine and prescribe lighter and faster JS loads, which seem to be essential to improve the athlete’s ability to apply force at higher movement velocities [4, 6, 23, 24]. It is worth noting that the mechanical differences between JS and HS decrease progressively with increasing load, which may compromise, or at least reduce, the “potential advantages” (i.e., greater force and power outputs at light-to-moderate loads) of ballistic exercises [6, 8, 23]. In this regard, the use of the appropriate JS-1RM load together with the reference bar-velocity values and equations provided herein (Table 2) is highly recommended, especially in high-performance training settings.

In summary, the load-velocity relationship in the JS is stable and consistent, even when including very-light loads (i.e., ~20% JS-1RM) in the predictive models. Among mean and peak velocity measures, PV appears as the most reliable and sensitive parameter [25]; nonetheless, all bar-velocity outputs have acceptable reliability, as indicated by their low CVs (≤ 5%) and high ICCs (≥ 0.90). Hence, any of these reference values can be accurately used to determine relative loads in the JS exercise. Future studies are required to examine and compare the effects of performing loaded JS under this novel loading strategy (i.e., determining JS loads from the load-velocity relationship based on the JS-1RM).

## CONCLUSIONS

We tested and confirmed the stability of the load-velocity relationship in the JS exercise, from very-light loading conditions (i.e., ~20% JS-1RM) up to the JS-1RM (i.e., ~86% HS-1RM) [8]. Coaches and sport scientists can utilize the equations and reference values provided here to precisely prescribe and monitor JS loads during different training phases, bearing in mind that this “novel loading strategy” will preserve the mechanical characteristics of the ballistic JS, thus potentially optimizing neuromuscular training adaptations (i.e., ability to apply force at high velocities) [8].

## Conflict of interest declaration

The authors declared no conflict of interest.
